# Comparison of toxin removal outcomes in online hemodiafiltration and intra-dialytic exercise in high-flux hemodialysis: A prospective randomized open-label clinical study protocol

**DOI:** 10.1186/1471-2369-13-156

**Published:** 2012-11-23

**Authors:** Vaibhav Maheshwari, Lakshminarayanan Samavedham, Gade Pandu Rangaiah, Yijun Loy, Lieng Hsi Ling, Sunil Sethi, Titus Lau Wai Leong

**Affiliations:** 1 Department of Chemical and Biomolecular Engineering, National University of Singapore, Singapore, Singapore; 2 Rehabilitation Medicine, National University Hospital, Singapore, Singapore; 3 National University Heart Center, Singapore, Singapore; 4 Yong Loo Lin School of Medicine, National University of Singapore, Singapore, Singapore; 5 Department of Laboratory Medicine, National University Hospital, Singapore, Singapore; 6 Division of Nephrology, Department of Medicine, National University Hospital, Singapore, Singapore

**Keywords:** Hemodialysis, Hemodiafiltration, Intra-dialytic exercise, Toxin removal, Inter-compartmental resistance, Cardiac output, Regional blood flow model, Spent dialysate, Blood temperature

## Abstract

**Background:**

Maintenance hemodialysis (HD) patients universally suffer from excess toxin load. Hemodiafiltration (HDF) has shown its potential in better removal of small as well as large sized toxins, but its efficacy is restricted by inter-compartmental clearance. Intra-dialytic exercise on the other hand is also found to be effective for removal of toxins; the augmented removal is apparently obtained by better perfusion of skeletal muscles and decreased inter-compartmental resistance. The aim of this trial is to compare the toxin removal outcome associated with intra-dialytic exercise in HD and with post-dilution HDF.

**Methods/design:**

The main hypothesis of this study is that intra-dialytic exercise enhances toxin removal by decreasing the inter-compartmental resistance, a major impediment for toxin removal. To compare the HDF and HD with exercise, the toxin rebound for urea, creatinine, phosphate, and β_2_-microglobulin will be calculated after 2 hours of dialysis. Spent dialysate will also be collected to calculate the removed toxin mass. To quantify the decrease in inter-compartmental resistance, the recently developed regional blood flow model will be employed. The study will be single center, randomized, self-control, open-label prospective clinical research where 15 study subjects will undergo three dialysis protocols (a) high flux HD, (b) post-dilution HDF, (c) high flux HD with exercise. Multiple blood samples during each study session will be collected to estimate the unknown model parameters.

**Discussion:**

This will be the first study to investigate the exercise induced physiological change(s) responsible for enhanced toxin removal, and compare the toxin removal outcome both for small and middle sized toxins in HD with exercise and HDF. Successful completion of this clinical research will give important insights into exercise effect on factors responsible for enhanced toxin removal. The knowledge will give confidence for implementing, sustaining, and optimizing the exercise in routine dialysis care. We anticipate that toxin removal outcomes from intra-dialytic exercise session will be comparable to that obtained by standalone HDF. These results will encourage clinicians to combine HDF with intra-dialytic exercise for significantly enhanced toxin removal.

**Trial registration:**

ClinicalTrials.gov number, NCT01674153

## Background

Hemodialysis is a life saving treatment for end stage renal disease (ESRD) patients and is prescribed to more than one million patients world-wide. Patients on dialysis are often associated with lower quality of life (QoL), significant burden of cardiovascular diseases, and numerous other co-morbidities [1]. Advances in hemodialysis care has resulted in improved patient outcomes and nearly 25% decline in mortality is observed in the last two decades, nonetheless the high rate of all-cause mortality in the early months of therapy is a matter of concern. Quantitatively, only 51% of dialysis patients are still alive three years after their initiation into renal replacement therapy. Mortality in the ESRD population is still 10 times greater than age standardized population without kidney failure [2]. Hence, much needs to be done to further improve patients’ outcomes.

Numerous aspects such as increasing uremic toxin removal, achieving patient dry weight or optimal fluid removal, prevention of intra-dialytic hypotensive episodes, maintaining optimal hemoglobin level, lowering phosphate level, precise electrolytes balance, and reducing incidence of hypertension, can be considered for improving the hemodialysis care. However, the plausible reason for undesired patient outcomes is insufficient removal of accumulated toxins, which over time lead to toxin overload followed by life deteriorating complications. Hence, ways to improve the toxin clearance forms the basis for this clinical research. The solute removal can be augmented by increasing the blood flow, dialysate flow, larger dialyzer, increase in dialysis time and/or frequency, or increasing the toxin removal by convection based renal replacement therapies (RRTs). Increasing the dialysis time and frequency corresponds to changing the conventional 4 hours × 3 times a week hemodialysis regimen to long nocturnal dialysis, daily short dialysis, respectively. However, many patients find it difficult and are reluctant to change from the traditional hemodialysis scheduling, as they have long established their living pattern to conventional dialysis. Dialysis care centers have also largely built their business model on the 4 hourly 3 times a week schedule. Hence, ways to improve the toxin clearance during the usual 4 hours of hemodialysis forms the basis of this clinical research.

In this regard, the convection based renal replacement therapy – hemodiafiltration (HDF) has been a subject of major research [3], and various randomized controlled trials have proved its efficacy for toxin removal [4-8]. The basic premise for encouraging HDF is the forced ultrafiltration rate that results in increased removal of middle or large sized toxins via convection [9,10]. These middle or large sized toxins have been shown to be associated with increased mortality and morbidity, and their increased removal may lead to better patient outcomes [11-13]. One common marker for these large sized toxins is β_2_-microglobulin. It is relatively easy to measure and its removal kinetics has been studied extensively [14-16].

Despite numerous documented benefits of HDF, there is still lack of evidence from various clinical trials, that HDF can improve the mortality and reduce morbidity, thus there is a need of properly designed randomized controlled clinical trials [17,18]. A prospective clinical study comparing online-HDF and high flux HD has shown that both small (urea and creatinine) and large (β_2_-microglobulin and complement factor D) sized toxin removal were greater for HDF when compared to high-flux HD. However, this increased removal of urea and creatinine did not result in lower pre-treatment serum concentration in both groups. In the context of large sized molecules, the authors found that, after one year, pre-treatment serum β_2_-microglobulin levels were similar in both regimens, but significant decrease was observed for complement factor D [5]. Based on these evidences, it was concluded that efficacy of HDF is largely restricted by inter-compartmental resistance [15]. Toxins, which are distributed in intracellular and interstitial compartments, in addition to blood plasma compartment, are severely restricted by cellular membrane or capillary endothelium. This also explains the significant removal of complement factor D, for its distribution is limited to intravascular compartment only. But majority of uremic toxins are distributed in both intracellular and extracellular compartment, and thus HDF or high-flux HD seem to detoxify the blood plasma compartment primarily. Hence, it is more important to focus on patient physiology i.e. overcoming the inter-compartmental resistance rather than focusing on improvement of RRTs. Above arguments also led to conclude that, after a certain volume of replacement fluid in HDF, there will not be any significant benefit for toxin removal, as blood plasma compartment will almost be devoid of toxins and inflow of toxins from remote compartments is restricted by inter-compartmental resistance. This explains why even with very high volume of replacement fluid (60L), there was no significant improvement in toxin removal [7], and the only plausible but not widely recommended way to overcome this resistance is to wait i.e. prescribe long nocturnal dialysis or increased frequency of dialysis, which have been found to be more efficacious than the high flux hemodialysis [19,20]. In another clinical study for 20 subjects, where removal outcomes of HDF and low flux HD were compared for asymmetric dimethylarginine (ADMA); there was no benefit from HDF over HD in lowering the ADMA concentration, rather authors found that HD was superior in increasing the L-arginine/ADMA ratio [21]. The reason for insignificant ADMA removal was associated with protein binding of this compound [21], however, ADMA is metabolic by-product of protein modification process in human cells, thus it may also be chiefly intra-cellular, and so HDF will be inefficient for its removal. This further strengthens the conclusion of Ward *et al*. that inter-compartmental resistance is the major barrier for toxin removal [15]. The HDF superiority over conventional high-flux HD in long term clinical trials is still debated [18], and we do not intend to delve on this debate. Rather, our intention is – how can we further improve the toxin removal performance of HDF by overcoming the physiological barrier.

Exercise during dialysis or intra-dialytic exercise has shown its potential for removal of uremic toxins in a number of clinical trials [1,22-25]. The increased toxin removal due to intra-dialytic exercise is ascribed to increased perfusion of skeletal muscles which results in mobilization of toxins from remote inaccessible compartments to intravascular compartment. It has been postulated that intra-dialytic exercise increases the cardiac output (CO), and the major portion of this increased CO is rendered to the low flow region (LFR) comprising skin, muscles, and bones, which contains almost 80% of body fluid and proportional amount of toxins. In normal condition i.e. no exercise scenario, only 20% of CO perfuses the LFR, and the rest 80% goes to high flow region (HFR) comprising heart, liver, kidney, brain, and blood itself, containing only 20% of bodily fluid and proportional amount of toxins. Based on this physiological disproportion of CO distribution, Schneditz *et al*. proposed the regional blood flow (RBF) model and explained the post-dialytic rebound phenomena for urea and creatinine [26,27], Smye *et al*. first employed this RBF model for explaining the effect of intra-dialytic exercise with the assumption of increased CO [28]. However, in our recent simulations for explaining the effect of intra-dialytic exercise using developed diffusion adjusted RBF (DA-RBF) model for β_2_-microglobulin [14], we observed that increase in CO due to exercise alone is insufficient to explain the increased toxin removal or reduced post-dialytic rebound. Hence, it was hypothesized that intra-dialytic exercise not only increases CO but also significantly decreases the inter-compartmental resistance, which is suggested as major barrier for inter-compartmental toxin transfer and for reduced HDF efficacy [14,15]. The inter-compartmental resistance is quantified by inversely proportional model parameter, named as inter-compartmental mass transfer coefficient; however, direct measurement of this model parameter is not possible. The indirect measurement or here parameter estimation will be performed via toxin kinetic modeling with repeated blood samples during and after dialysis. Hence, in this clinical research, we will study the effect of intra-dialytic exercise in conventional dialysis settings and quantify the associated physiological changes responsible for enhanced toxin removal. Additionally, no clinical study has investigated the effect of intra-dialytic exercise for removal of middle molecules toxin marker, β_2_-microglobulin. It has increasingly been considered as marker of toxin milieu; hence effect of intra-dialytic exercise on β_2_-microglobulin removal will also be considered in this clinical research [29,30].

### Specific aims

1. Investigate the effect of intra-dialytic exercise on removal of middle molecule marker toxin, β_2_-micorglobulin and compare the same with conventional hemodialysis session.

2. Investigate the effect of intra-dialytic exercise on physiological changes, namely, decrease in inter-compartmental resistance, increase in CO, change in heart rate and blood pressure via direct measurement and indirect measure i.e. toxin kinetic modeling.

3. Compare the toxin and solute removal outcomes for urea, creatinine, phosphate, β_2_-micorglobulin, sodium, and potassium for post-dilution HDF and intra-dialytic exercise in conventional hemodialysis setting using spent dialysate collection as well as post-dialysis rebound calculation.

## Methods and design

### Study design and setting

This study is single center, open label, self-controlled (within subject design), randomized prospective, efficacy study involving patients undergoing conventional high-flux hemodialysis. Blinding is not feasible, for the changes in conventional HD are immediately visible to both study subjects and investigators. The study will be conducted at outpatient dialysis center of National University Hospital (NUH), Singapore.

### Ethics approval and quality assurance

The domain specific review board affiliated with National Healthcare Group (NHG), Singapore has approved the trial. The study will undergo routine quality assurance review conducted by the ethics board. The ethics board will also receive timely progress status report from the principal investigator and will be promptly informed of any adverse events owing to the study protocol.

### Patient recruitment

The principal investigator and study administrator will review the existing patients’ database for hemoglobin level and ejection fraction on prior test results, existing chronic obstructive pulmonary disease (COPD), angina, and history of heart-attack, before contacting the patients. The study subjects should have no residual renal function (defined by urine output of less than 200 mL/day). Potential subjects satisfying the above mentioned criteria will be identified and subsequently contacted for preliminary tests where patients will be explained about the study protocol, and benefits of HDF and intra-dialytic exercise. Agreed patients satisfying the inclusion–exclusion criteria will sign the patient information sheet and consent form in the presence of an independent witness, who will also sign the form. A copy of signed form will be given to the patient. Total 15 subjects will be recruited for the study.

#### Inclusion criteria

1. Adult patients male or female (Age > 21 years).

2. Minimum dialysis vintage of 2 months.

3. Stable on hemodialysis.

4. Minimum Hemoglobin level of 10 g/dL.

5. Blood access capable of delivering the blood flow rate greater than 250 mL/min.

6. Preserved left ventricular ejection fraction (>50%) on prior imaging study.

7. Desirable performance in 6 min-walk-test (6 MWT).

#### Exclusion criteria

1. History of recurring or persistent hypotension in the past 2 months.

2. Pregnant woman.

3. Severely Hypertensive patients (systolic blood pressure > 180 mmHg and/or diastolic blood pressure > 115 mmHg).

4. History of recent myocardial infarction or unstable angina (within the past 6 months).

5. Significant valvular disease, i.e. severe aortic stenosis and moderate-severe mitral regurgitation.

6. Patients with end stage organ disease e.g. COPD, recent or debilitating CVA.

7. Patient with recent stroke (within the past 6 months).

8. Anemic patients.

9. History of known arrhythmia.

10. Participation in another clinical intervention trial.

11. Moderate to severe osteoarthritis of knee(s).

12. Unable to consent.

The consented subjects will be called for 6 MWT, which will be performed according to the standard guidelines prescribed by American Thoracic Society [31]. Patients will walk along a measured circuit (30 m), instructed to cover as much distance as they can within 6 min. Blood pressure (BP), heart rate (HR) and rate of perceived exertion (RPE) will be assessed at pre- and post- 6 MWT. HR and RPE will be measured at every minute of the test, as well as at 8 and 10 min to assess heart rate recovery (HRR) and blood pressure. This test has been considered as appropriate sub-maximum test to assess patient’s functional and physiological response, cardiovascular fitness, and suitability for intra-dialytic exercise in ESRD population [32]. Patients who ambulate less than 300 m over 6 min will be excluded from the test due to the likelihood that they may not tolerate the exercise protocol (described later). Patient should also demonstrate acceptable physiological response during the test (e.g. HRR, hemodynamic parameters within safe guidelines for exercise.

### Study interventions and randomization

All recruited study subjects will undergo three different study dialysis sessions, namely, HD, HDF, and HD with exercise within a maximum time interval of 6 months between the first and last study session. High flux HD will be conducted as per the patient routine dialysis session; HDF will be conducted with 18L of replacement fluid. Patients’ medication (phosphate binder, medication for hypertension, erythropoietin dose, etc.) will not be changed during the study. Minimum one week gap will be maintained between study sessions for each individual patient. This will remove any carry-over effect of previous study session and will bring the patients to their nominal toxin concentration level. Patients will also be advised to keep their diet fairly constant during the study period. The study sessions will be conducted in mid-week or end-week setting; however, study sessions for an individual subject will be on the same day of the week. All sessions will be conducted in the randomized order and random sequence of study sessions will be generated using a computer, though patient’s existing dialysis schedule will not be disturbed. To avoid the effect of confounding factors, same dialysis conditions will be used in all three dialysis sessions.

The intra-dialytic exercise will be conducted in conventional high-flux dialysis setting with exercise prescription in three bouts. The first exercise bout will be from 40-60 min, second from 80-100 min, and third from 120-140 min. If patients are unable to adhere to the prescribed exercise intervention then patients will be advised to perform a total of 60 min exercise during the dialysis. The exercise will be sustained till the achievement of 60% of maximum heart rate, and will be given via active cycling movement (ACM) using calibrated Monark 881E cycle ergometer (Monark, Sweden). Various hemodynamic responses viz. heart rate, blood pressure, arterial and venous blood temperature will be measured during all the study sessions. Additional hemodynamic information viz. cardiac output, peripheral vascular resistance index, etc. will also be obtained using Doppler Echocardiography, and will be made before the onset of dialysis, before starting the exercise, at 5-minute intervals during exercise, and after termination of first exercise bout. Echocardiography will also be used to assess cardiac structure and function before and during exercise – any new or unexpected abnormality, e.g. regional dysfunction, will be highlighted to the dialysis team.

### Data collection

#### Blood and dialysate sampling

Total 10 arterial blood samples, 6 during and 4 after dialysis will be collected in each study session. Each blood sample will be analyzed for uremic toxins, namely, urea, creatinine, phosphate, β_2_-microglobulin, and uremic solutes, namely, sodium and potassium. The volume of each collected blood sample will be 4 mL. An additional post-dialyzer i.e. venous blood sample at 60 min will be collected to calculate the dialyzer clearance for the above mentioned toxins/solutes. All the blood samples will be sent to NUH clinical laboratory immediately after collection. These toxins/solutes concentrations will be used for estimating the parameters of the recently developed diffusion-adjusted regional blood flow model, which will characterize the physiological changes due to HD, intra-dialytic exercise, and standalone HDF. The validity of model will be assessed for urea, creatinine, phosphate, and β_2_-microglobulin.

Collection of total spent dialysate is the ‘gold standard’ for measuring the amount of toxin removed. However, the volume of total spent dialysate will be 120 L for 500 mL/min dialysate flow rate and will be 20 L more in case of HDF; the volume of spent dialysate will be further augmented by fluid removed from the patient. This will require a large container and space which is generally not available in the dialysis center. Hence, a representative fraction of spent dialysate will be collected to assess the quantum of toxin removed. This approach has already been validated for quantifying the amount of urea removed [33]. In total, 45 study dialysis sessions will be conducted; where a total of 450 arterial and 45 venous blood samples will be collected; a total of 45 dialysate samples will be analyzed.

#### Blood temperature monitoring

Arterial and venous blood temperature will be continuously monitored using blood temperature monitor (Fresenius, Bad Homburg, Germany). It is hypothesized that intra-dialytic will increase the body core temperature. Note that, the dialysate temperature will be kept same for all three study sessions. All the collected samples and data will be assigned a unique study number, which will be used to maintain patient confidentiality.

### Treatment modification

All therapies will be discontinued, should adverse-effects such as acute hypotensive episode (defined by blood pressure drop of 20 mm Hg or SBP < 85) or chest pain, uneasiness or cramps during study session manifest. And the session will be resumed only after patient stabilizes. Appropriate intervention will be prescribed to stabilize the subject. Although these are anticipated to be at low event occurrence rate, the event will be medically recorded and dealt with following the usual clinical practice. A report of all adverse events will be provided to the ethics board.

### Outcome measures

#### Primary outcome measure

A representative fraction of spent dialysate will be analyzed to quantify the removed toxin mass in each study dialysis session viz. HD, HDF, and HD with exercise. The removed toxin mass for standalone HDF and HD with exercise will be compared using standard statistical *t*-test. The post-dialytic rebound will also be quantified using pre-dialysis (at t = 0 min), end-dialysis (at t = 240 min), and post-dialysis (at t = 360 min) toxin concentration.

#### Secondary outcome measure

**Figure 1:**
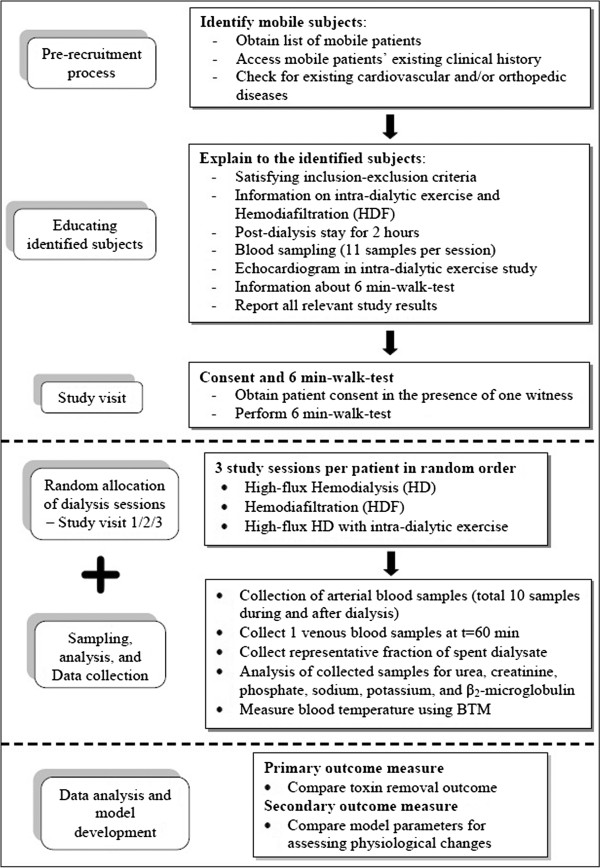
Flow chart of the clinical trial.

The blood toxin(s) concentration will be employed to estimate model parameters for individual toxin in each individual session. One of the estimated model parameters (inter-compartmental mass transfer coefficient) will quantify the physiological changes responsible for enhanced toxin removal due to intra-dialytic exercise. The change in estimated parameter value will be compared using standard *t*-test. The conventional HD session will be treated as control. Increase in cardiac output and decrease in peripheral vascular resistance index will also be inferred from Doppler echocardiography during intra-dialytic exercise study. The schematic flow chart of clinical trials is presented in Figure 1.

## Discussion

Intra-dialytic exercise was first studied by Painter *et al*. in prospective clinical trials for routine outpatient hemodialysis patients [34]. Since then, exercise has been advocated as adjunctive intervention for maintenance HD patients and numerous benefits have been accounted in clinical literature. Despite the documented benefits, nephrologists as well as patients are not enthusiastic to accommodate exercise in routine dialysis care, and still it is considered as intervention rather than a routine care. One of the reasons behind this is requirement of individualized prescription for intra-dialytic exercise [35]. Before we aim for individualized prescription, it is important to understand how intra-dialytic exercise brings in physiological changes responsible for enhanced toxin removal.

**Figure 2:**
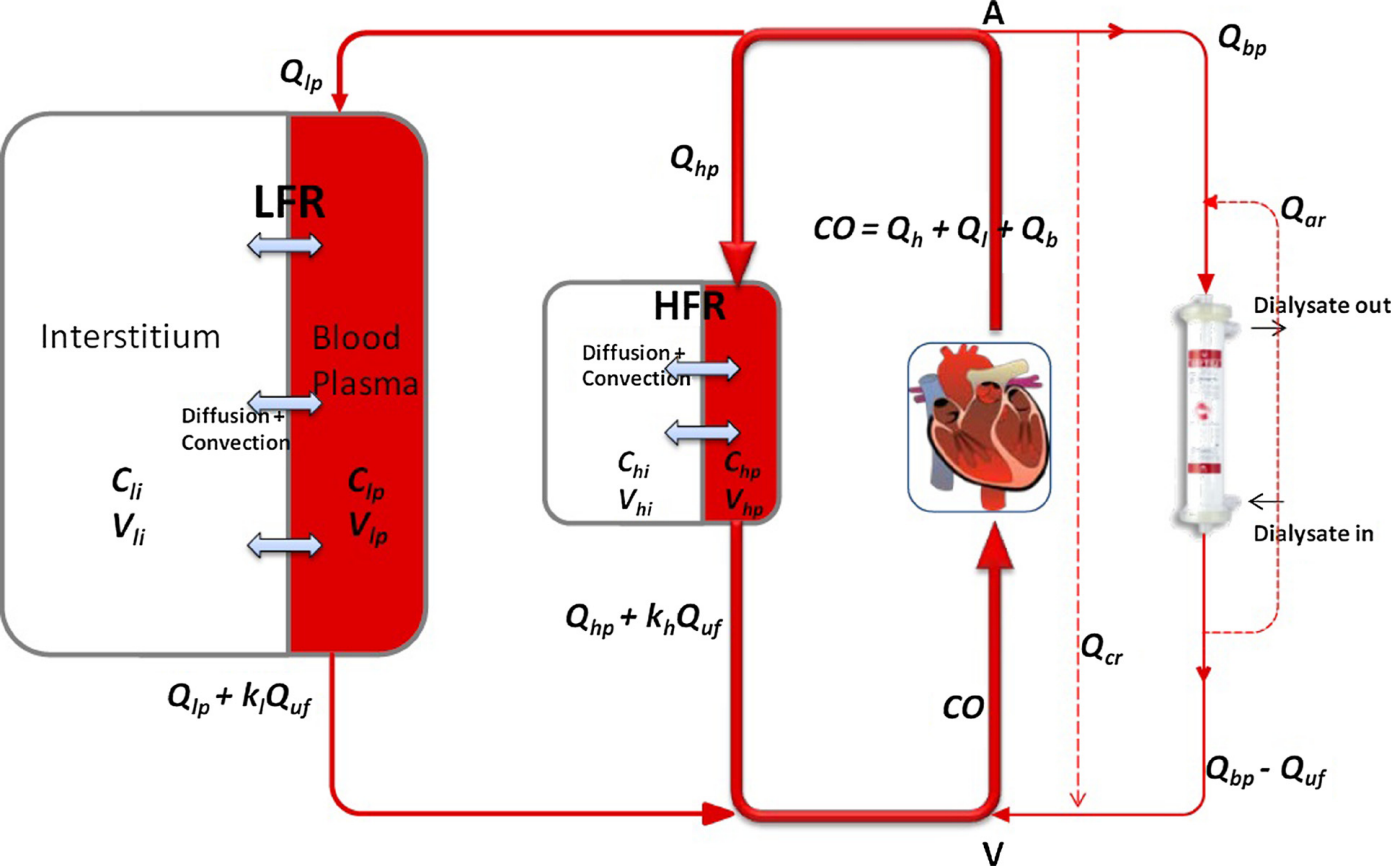
**Diffusion-adjusted regional blood flow model (parallel-cum-series representation of physiology) or explaining β_2_-microglobulin kinetics.** Toxin transfer is due to diffusion across capillary endothelium, and blood/plasma circulation causes convective transport. *Q*_h_/*Q*_hp_, *Q*_l_/*Q*_lp_, and *Q*_b_/*Q*_bp_ are blood/plasma flows to high flow region (HFR), low flow region (LFR), and dialyzer, respectively. *Q*_cr_ and *Q*_ar_ are cardiopulmonary and access recirculation, respectively. Shaded compartments represent contact with blood (A – arterial node and V – venous node) [14].

A number of physiological changes have been speculated by researchers. Parson *et al*. have suggested that increased cardiac output and thus increased blood flow to lower extremities and open capillary surface area would increase the flux of toxins from tissue to vascular compartment [1]. Recently, using mathematical simulations of validated model, it was hypothesized that intra-dialytic exercise not only increases the cardiac output but also results in significant decrease in inter-compartmental resistance owing to capillary endothelium or cellular membrane. One can infer cardiac output using available devices such as Transonic flow-QC system or by Doppler Echocardiogram, but it is not possible to measure inter-compartmental resistance directly, as it is resistance due to membrane/capillary endothelium, and thus it was quantified using inter-compartmental mass transfer coefficient which is inversely proportional to inter-compartmental resistance. The inter-compartmental mass transfer coefficient is one of the model parameters which will be estimated using collected blood samples. The increase in the estimated parameter will give quantitative validity of intra-dialytic exercise over conventional dialysis. The physiological representation of regional blood flow model for middle molecular marker toxin is shown is Figure 2. For more details on model and corresponding model equations, readers are advised to refer original paper [14], and paper by Schneditz et al. who developed the diffusion-adjusted regional blood flow model for small-sized toxins [26].

Apart from discussed physiological changes i.e. increased cardiac output and increased inter-compartmental mass transfer coefficient, it is hypothesized that intra-dialytic exercise will increase the body core temperature which will probably further dilate the vasculature. This assumption is based on the clinical study by Kalousová et al. where effect of cool dialysate temperature was studied on hemodynamic stability and urea kinetics. Authors noted that for less than 1°C decrease in body core temperature (due to cool dialysate), there was corresponding ~400% increase in peripheral vascular resistance index [21], but this did not affect the urea removal. However, caution should be exercised before generalizing the authors’ conclusion for all uremic toxins, because urea removal is primarily flow controlled i.e. with increased blood flow urea clearance increases [26], while removal of other middle sized toxins or even smaller toxins like creatinine is primarily restricted by inter-compartmental resistance [15,26]. Hence, with increase in peripheral vascular resistance index, the removal of such compartment resistance controlled toxins will be significantly curbed. As exercise will plausibly increase the body core temperature, and hence decrease the peripheral vascular resistance, it will result in increased toxin removal from remote inaccessible compartments. To the best of our knowledge, there is no clinical study where the effect of intra-dialytic exercise on body core temperature has been studied. To confirm our hypothesis, we will measure the change in arterial blood temperature in all sessions. It has also been recommended that applicability of DA-RBF model should be assessed for small as well as large sized toxins, but this should be performed where marker toxins are measured and analyzed within the same experiment [36]. The proposed clinical study intends to fill that gap.

Finally, it is important to test the efficacy of intra-dialytic exercise in mobilization of toxins from remote inaccessible compartments to plasma compartment. Subsequent removal of the mobilized toxins from plasma compartment should be accomplished by an efficient renal replacement therapy like HDF. Hence, the positive outcomes of this clinical trial will encourage clinicians to combine HDF with intra-dialytic exercise; thereby obtaining the maximum possible toxin removal with significant reduction in post-dialysis toxin rebound.

## Abbreviations

HD: Hemodialysis; HDF: Hemodiafiltration; CO: Cardiac output; ESRD: End Stage Renal disease.

## Competing interest

Authors declare that there is no competing interest.

## Authors’ contribution

VM conceived and designed the research protocol, and wrote the manuscript. LS, GPR, and TLWL have given valuable inputs for improvement of mathematical model, study protocol, and blood sampling. YL has suggested important aspects for exercise protocol, LLH has contributed in patients’ inclusion–exclusion criteria and analyzing various hemodynamic responses, while SS has assisted in blood and dialysate sampling protocol. All authors have contributed for writing this manuscript.

## Pre-publication history

The pre-publication history for this paper can be accessed here:

http://www.biomedcentral.com/1471-2369/13/156/prepub

## References

[B1] Parsons TL Toffelmire EB King-Van Vlack CE Exercise training during hemodialysis improves dialysis efficacy and physical performance Arch Phys Med Rehabil 2006 87 680 687 10.1016/j.apmr.2005.12.044 16635631

[B2] U S Renal Data System, USRDS 2012 Annual Data Report Atlas of Chronic Kidney Disease and End-Stage Renal Disease in the United States, National Institutes of Health 2012 Bethesda, MD: National Institute of Diabetes and Digestive and Kidney Diseases

[B3] Krick G Ronco C On-line Hemodiafiltration: The Journey and the Vision. S Karger Ag 2011

[B4] Schiffl H Prospective randomized cross-over long-term comparison of online haemodiafiltration and ultrapure high-flux haemodialysis Eur J Med Res 2007 12 26 33 17363355

[B5] Ward RA Schmidt B Hullin J Hillebrand GF Samtleben W A comparison of on-line hemodiafiltration and high-flux hemodialysis: A prospective clinical study J Am Soc Nephrol 2000 11 2344 2350 11095657 10.1681/ASN.V11122344

[B6] Pedrini LA De Cristofaro V Comelli M Casino FG Prencipe M Baroni A Campolo G Manzoni C Colì L Ruggiero P Long-term effects of high-efficiency on-line haemodiafiltration on uraemic toxicity, A multicentre prospective randomized study Nephrol Dial Transplant 2011 26 2617 2624 10.1093/ndt/gfq761 21245130

[B7] Wizemann V Lotz C Techert F Uthoff S Online haemodiafiltration versus low-flux haemodialysis, A prospective randomized study Nephrol Dial Transplant 2000 15 43 48 10737166 10.1093/oxfordjournals.ndt.a027963

[B8] Maduell F Arias M Vera M Fontseré N Blasco M Barros X Garro J Elena M Bergadá E Cases A Mid-dilution hemodiafiltration: A comparison with pre- and postdilution modes using the same polyphenylene membrane Blood Purif 2009 28 268 274 10.1159/000232935 19684394

[B9] Thomas G Jaber BL Convective therapies for removal of middle molecular weight uremic toxins in end-stage renal disease: a review of the evidence Semin Dial 2009 22 610 614 10.1111/j.1525-139X.2009.00665.x 20017830

[B10] Locatelli F Manzoni C Cavalli A Di Filippo S Can convective therapies improve dialysis outcomes? Curr Opin Nephrol Hypertens 2009 18 476 480 10.1097/MNH.0b013e3283318e8b 19726986

[B11] Drueke TB β2-Microglobulin and amyloidosis Nephrol Dial Transplant 2000 15 17 24 10.1093/oxfordjournals.ndt.a027958 10737162

[B12] Cheung AK Rocco MV Yan G Leypoldt JK Levin NW Greene T Agodoa L Bailey J Beck GJ Clark W Serum beta-2 microglobulin levels predict mortality in dialysis patients: Results of the HEMO study J Am Soc Nephrol 2006 17 546 555 10.1681/ASN.2005020132 16382021

[B13] Okuno S Ishimura E Kohno K Fujino-Katoh Y Maeno Y Yamakawa T Inaba M Nishizawa Y Serum beta2-microglobulin level is a significant predictor of mortality in maintenance haemodialysis patients Nephrol Dial Transplant 2009 24 571 577 18799606 10.1093/ndt/gfn521

[B14] Maheshwari V Samavedham L Rangaiah G A Regional Blood Flow Model for β2-Microglobulin Kinetics and for Simulating Intra-dialytic Exercise Effect Ann Biomed Eng 2011 39 2879 2890 10.1007/s10439-011-0383-5 21877220

[B15] Ward RA Greene T Hartmann B Samtleben W Resistance to intercompartmental mass transfer limits beta(2)-microglobulin removal by post-dilution hemodiafiltration Kidney Int 2006 69 1431 1437 16395268 10.1038/sj.ki.5000048

[B16] Stiller S Xu XQ Gruner N Vienken J Mann H Validation of a two-pool model for the kinetics of Beta 2-microglobulin Int J Artif Organs 2002 25 411 420 12074339 10.1177/039139880202500511

[B17] Locatelli F Comparison of Hemodialysis, Hemodiafiltration, and Hemofiltration: Systematic Review or Systematic Error? Am J Kidney Dis 2005 46 787 788 16183439 10.1053/j.ajkd.2005.06.026

[B18] Blankestijn PJ Ledebo I Canaud B Hemodiafiltration: clinical evidence and remaining questions Kidney Int 2010 77 581 587 10.1038/ki.2009.541 20130529

[B19] Mucsi I Hercz G Uldall R Ouwendyk M Francoeur R Pierratos A Control of serum phosphate without any phosphate binders in patients treated with nocturnal hemodialysis Kidney Int 1998 53 1399 1404 10.1046/j.1523-1755.1998.00875.x 9573558

[B20] Eloot S van Biesen W Dhondt A de Smet R Marescau B De Deyn PP Verdonck P Vanholder R Impact of increasing haemodialysis frequency versus haemodialysis duration on removal of urea and guanidino compounds: a kinetic analysis Nephrol Dial Transplant 2009 24 2225 2232 10.1093/ndt/gfp059 19225018

[B21] Kalousová M Kielstein JT Hodková M Zima T Dusilová-Sulková S Martens-Lobenhoffer J Bode-Boger SM No Benefit of Hemodiafiltration over Hemodialysis in Lowering Elevated Levels of Asymmetric Dimethylarginine in ESRD Patients Blood Purif 2006 24 439 444 10.1159/000095360 16940714

[B22] Giannaki CD Stefanidis I Karatzaferi C Liakos N Roka V Ntente I Sakkas GK The Effect of Prolonged Intradialytic Exercise in Hemodialysis Efficiency Indices ASAIO J 2011 57 213 218 10.1097/MAT.0b013e318215dc9e 21412149

[B23] Vaithilingam I Polkinghorne KR Atkins RC Kerr PG Time and exercise improve phosphate removal in hemodialysis patients Am J Kidney Dis 2004 43 85 89 10.1053/j.ajkd.2003.09.016 14712431

[B24] Kong CH Tattersall JE Greenwood RN Farrington K The effect of exercise during haemodialysis on solute removal Nephrol Dial Transplant 1999 14 2927 2931 10.1093/ndt/14.12.2927 10570099

[B25] Farese S Budmiger R Aregger F Bergmann I Frey F Uehlinger D Effect of transcutaneous electrical muscle stimulation and passive cycling movements on blood pressure and removal of urea and phosphate during hemodialysis Am J Kidney Dis 2008 52 745 752 10.1053/j.ajkd.2008.03.017 18487001

[B26] Schneditz D Platzer D Daugirdas JT A diffusion-adjusted regional blood flow model to predict solute kinetics during haemodialysis Nephrol Dial Transplant 2009 24 2218 2224 10.1093/ndt/gfp023 19211646

[B27] Schneditz D Van Stone JC Daugirdas JT A Regional blood circulation alternative to in-series two compartment urea kinetic modeling ASAIO J 1993 39 M573 M577 8268602

[B28] Smye S Lindley E Will E Simulating the effect of exercise on urea clearance in hemodialysis J Am Soc Nephrol 1998 9 128 132 9440097 10.1681/ASN.V91128

[B29] Vanholder R Eloot S Van Biesen W Do we need new indicators of dialysis adequacy based on middle-molecule removal? Nat Clin Pract Nephrol 2008 4 174 175 10.1038/ncpneph0750 18268526

[B30] Drüeke TB Massy ZA Progress in uremis toxin research: Beta2-microglobulin Semin Dial 2009 22 378 380 10.1111/j.1525-139X.2009.00584.x 19708985

[B31] Statement ATS Guidelines for the Six-Minute Walk Test Am J Respir Crit Care Med 2002 166 111 117 12091180 10.1164/ajrccm.166.1.at1102

[B32] Fitts SS Guthrie MR Six-minute walk by people with chronic renal failure: assessment of effort by perceived exertion Am J Phys Med Rehabil 1995 74 54 10.1097/00002060-199501000-00009 7873114

[B33] Yu A Wong F Rafiq M Zhou F Daugirdas J Collection of a representative fraction of total spent hemodialysate Am J Kidney Dis 1995 25 810 812 10.1016/0272-6386(95)90560-X 7747738

[B34] Painter PL Nelson-Worel JN Hill M Thornbery D Shelp W Harrington A Weinstein A Effects of exercise training during hemodialysis Nephron 1986 43 87 92 10.1159/000183805 3713951

[B35] Bennett PN Breugelmans L Barnard R Agius M Chan D Fraser D McNeill L Potter L Sustaining a hemodialysis exercise program: a review Semin Dial 2010 23 62 73 10.1111/j.1525-139X.2009.00652.x 20331819

[B36] Schneditz D F1000 Recommendation of [Maheshwari V et al Ann Biomed Eng 2011 39 12 2879 2890 Faculty of 1000, 27 Feb 2012. http://f1000.com/13830964#eval15270068 10.3410/f.13830964.15270068 21877220

